# A Novel Secretory Vesicle from Deer Antlerogenic Mesenchymal Stem Cell-Conditioned Media (DaMSC-CM) Promotes Tissue Regeneration

**DOI:** 10.1155/2018/3891404

**Published:** 2018-03-25

**Authors:** Minkoo Seo, Jin-Chul Kim, Hyung-Ki Kim, Eun wook Choi, Suyeong Jeong, Ki Chang Nam, Mihue Jang

**Affiliations:** ^1^Prostemics Research Institute, Seongdong-gu, Seoul, Republic of Korea; ^2^Natural Constituents of Research Center, Natural Products Research Institute, Korea Institute of Science and Technology (KIST), Seoul, Republic of Korea; ^3^Department of Medical Engineering, Dongguk University College of Medicine, Seoul, Republic of Korea; ^4^Center for Theragnosis, Biomedical Research Institute, Korea Institute of Science and Technology (KIST), Seoul, Republic of Korea

## Abstract

Multipotent stem cells have the capacity to generate terminally differentiated cell types of each lineage; thus, they have great therapeutic potential for a wide variety of diseases. The most widely available stem cells are derived from human tissues, and their use for therapeutic application is limited by their high cost and low productivity. Herein, we report that conditioned media of mesenchymal stem cells (MSCs) isolated from deer antlers enhanced tissue regeneration through paracrine action via a combination of secreted growth factors and cytokines. Notably, DaMSC-conditioned media (DaMSC-CM) enhanced hair regeneration by activating the Wnt signaling pathway. In addition, DaMSC-CM had regenerative potential in damaged skin tissue through induction of skin regeneration-related genes. Remarkably, we identified round vesicles derived from DaMSC-CM, with an average diameter of ~120 nm that were associated with hair follicle formation, suggesting that secretory vesicles may act as paracrine mediators for modulation of local cellular responses. In addition, these secretory vesicles could regulate the expression of Wnt-3a, Wnt-10b, and lymphoid enhancer-binding factor-1 (LEF-1), which are related to tissue renewal. Thus, our findings demonstrate that the use of DaMSC-CM as a unique natural model for rapid and complete tissue regeneration has possible application for therapeutic development.

## 1. Introduction

Adult mesenchymal stem cells (MSCs) are self-renewing progenitor cells. Multipotent stem cells have the capacity to generate terminally differentiated cell types of each lineage which can fabricate specific tissues, including skin, bone, cartilage, and adipose tissue. To date, it has been widely accepted that stem cells play significant roles in tissue repair and regeneration [1–4]. Stem cell-based regenerative therapies have been successfully developed, indicating that stem cells have great merit as a source of regenerative medicine [5–7]. However, harnessing stem cells for this purpose requires a better understanding of the mechanisms by which existing tissues affect stem cell-mediated regenerative action.

In addition to the multipotent differentiation potential of MSCs, several paracrine factors of MSCs have been discovered that contribute to modulation of tissue regeneration [8, 9]. MSCs can secrete bioactive molecules that act on cell migration, antioxidation, antiapoptosis, angiogenesis, and immunomodulation via regulation of local cellular responses [10–12]. Furthermore, several studies have demonstrated that injection of MSCs-CM also enhances tissue repair [13, 14]. The release of paracrine factors such as growth factors and cytokines can affect the stem cell microenvironment in response to tissue injury, which can activate tissue survival, repair, and regeneration. Remarkably, recent studies reported that vesicles derived from stem cells may be key mediators of stem cell paracrine mechanisms [15–19]. For example, adipose mesenchymal stem cell- (ASC-) derived extracellular vesicles can contribute to paracrine signaling [19]. Extracellular vesicles can be internalized by fibroblasts to promote cell migration, proliferation, and collagen synthesis. In addition, *in vivo* injection of extracellular vesicles into mice suffering from back wounds was shown to promote cutaneous wound healing by recruiting the vesicles to the damaged area. Thus, the multipotent potential of MSCs and paracrine mechanisms together affect repair and regeneration of damaged tissues.

Deer antlers are unique mammalian organs that undergo subsequent regrowth in response to seasonal changes throughout the animal's life and are thus a remarkably useful model of mammalian regeneration [20, 21]. It has been suggested that the annual renewal of deer antlers involves a stem cell-based process [22]. Antler regeneration might be initiated by self-renewing multipotent stem cells residing in the pedicle periosteum (PP), a permanent protuberance on the frontal bone. PP-derived cells express several stem cell markers, such as CD9, OCT-4, Nanog, and STRO-1, and exhibit pluripotency due to their distinctive regenerative ability to differentiate along several mesenchymal lineages *in vitro* [23, 24]. Nevertheless, the mechanism of regeneration in antlers remains unclear.

In this study, we demonstrated that conditioned media (CM) of cultured stem cells isolated from deer antlers play an important role in hair and skin regeneration by releasing paracrine factors that activate the Wnt signaling pathway. Furthermore, we discovered a novel mediator of paracrine actions in tissue regeneration, which indicates great potential for therapeutic application.

## 2. Methods

### 2.1. Isolation of Deer Antler-Derived MSCs (DaMSCs)

Fresh red deer (*Cervus elaphus*) antlers were obtained under conditions approved by the local Animal Ethics Committee and in accordance with protocols approved by the Institute Animal Care. Antlers were collected from anesthetized four-year-old red deer stags during late spring from a local deer farm (Han-Lim-Won, Korea). The antler tip, composed of a velvet skin, perichondrium, mesenchyme, and chondroprogeniors, was taken using sterilized surgical instruments and then mesenchyme was obtained by slices of tissues. The obtained samples were digested with 0.075% collagenase type II (Sigma-Aldrich, USA) under gentle agitation for 45 min at 37°C and then centrifuged at 300*g* for 10 min to obtain the stromal cell fraction. The pellets were filtered with a 70 mm nylon mesh and resuspended with phosphate-buffered saline (PBS). The solution was layered onto histopaque-1077 (Sigma-Aldrich, USA) and centrifuged at 840*g* for 10 min. The supernatant was discarded, and cell fraction was cultured overnight at 37°C, in Dulbecco's Modified Eagle media (DMEM) supplemented with 10% fetal bovine serum (FBS), 100 U/mL of penicillin, and 100 mg/mL of streptomycin.

### 2.2. Immunocytofluorescence

Immunocytofluorescence labeling was conducted on cultured DaMSCs. To detect stem cell markers, anti-STRO-1 antibodies (RnD Systems, USA) were used in combination with fluorescein isothiocyanate- (FITC-) conjugated anti-mouse IgM secondary antibodies (Molecular Probes, USA), and anti-CD90 antibodies (BD Biosciences, USA) were used in combination with Alexa Fluor 546-conjugated anti-mouse IgG secondary antibodies (Molecular Probes, USA). Primary antibodies were diluted 1 ∶ 50 and secondary antibodies 1 ∶ 100.

### 2.3. Protein Chip Assay

DaMSCs were incubated for 3 days in DMEM without FBS and then conditioned media of DaMSCs were harvested. After treatment of DaMSC-CM, a growth factor antibody array kit (Growth factor array C1, RayBio, USA) was accessed in according to the manufacturer's instructions. Normal media (DMEM) was treated as a negative control.

### 2.4. Cell Proliferation Assay

DaMSC-CM, ASC-CM, and normal media were used to treat three different types of cells including dermal papilla cells (DPCs), keratinocytes (HaCaTs) and human dermal fibroblasts (HDFs). At 48 h post incubation, a CCK-8 assay (Dojindo Molecular Technologies, USA) was conducted and cell proliferation was measured by absorbance at 450 nm using a microplate reader (Bio-Rad, USA).

### 2.5. Quantitative RT-PCR (qRT-PCR) Analysis

After 72 h treatment with DMEM, ASC-CM, or DaMSC-CM, total RNA extracts from three different types of cells (DPCs, HaCaTs, and HDFs) were obtained using an RNeasy Mini Kit (Qiagen, Germany). Each 1 *μ*g of total RNA was reverse transcribed using the iScriptTM cDNA Synthesis Kit (Invitrogen, Korea), and 50 ng of cDNA for each sample was used for qRT-PCR analysis (StepOne Real-Time PCR system, ABI) with the specific primers listed in Supplementary Table S1.

### 2.6. In Vitro Wound Healing Assay

For the measurement of cell migration, HDFs kept in serum-free media were placed onto a 60 mm dish to create a confluent monolayer and then wounding was generated with a plastic micropipette tip. After washing, the medium was replaced with control media or DaMSC-CM in a time-dependent manner. Photographs of wound spaces were taken by phase-contrast microscopy.

### 2.7. Isolation of Extracellular Vesicles (EVs) from DaMSC-CM

To isolate vesicles secreted from DaMSCs, conditioned media were collected. After collection of conditioned media, cell debris and large membrane particles were removed by sequential centrifugation at 500*g* for 30 min and then 10,000*g* for 30 min. Vesicles were collected after ultracentrifugation at 100,000*g* for 1 h and then resuspended in PBS for further experiments.

### 2.8. Characterization of EVs Isolated from DaMSC-CM

To analyze the size and morphology of EVs, nanoparticle tracking analysis (NTA) and transmission electron microscopy (TEM) analysis were performed, respectively. For measurement of particle concentrations, a NanoSight LM 10 instrument (NanoSight Ltd, Amesbury, UK) was used. For TEM images, vesicles fixed in 2% paraformaldehyde were transferred onto Formvar-carbon-coated electron microscopy grids. Grids were washed with PBS several times and covered with one drop of 1% glutaraldehyde for 5 min. After washing with distilled water, grids were negatively stained with 2% uranyl acetate and analyzed with a transmission electron microscope (JEM-1011, Germany).

## 3. Results

### 3.1. Identification of DaMSCs

**Figure 1:**
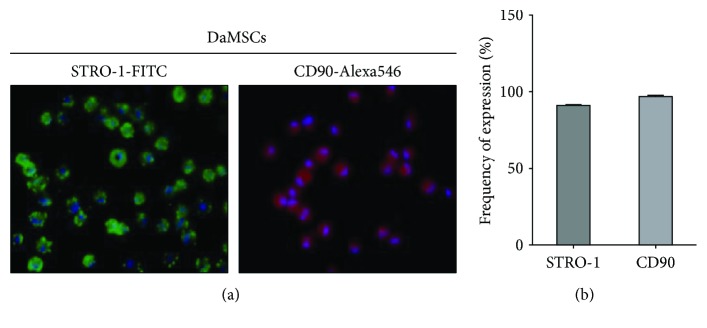
Immunolocalization of mesenchymal stem cell makers in deer antler-derived mesenchymal stem cells (DaMSCs). Immunocytochemistry staining was conducted on DaMSCs with primary antibodies directed against STRO-1 (a, left, green), and CD90 (a, right, red) and stained by FITC- and Alexa 546-conjugated secondary antibodies, respectively. Nuclei were visualized with DAPI (blue). (b) Graph indicates quantification of protein expression. Data represent the mean ± s.d. (*n* = 3).

To verify the presence of MSCs, red deer antler-derived MSCs were isolated using a sequential centrifugation approach. Immunohistochemistry assays were used to detect stem cell markers including STRO-1 and CD90 in DaMSCs (Figure 1). STRO-1-positive or CD90-positive MSCs were visualized using fluorescence microscopy (Figure 1(a)) and quantified by normalization with DAPI-stained nuclei (Figure 1(b)). In three randomly selected regions of a culture dish, >90% of observed cells were STRO-1-positive and >95% were CD90-positive.

### 3.2. Detection of Active Biomolecules from Conditioned Media of DaMSCs

**Figure 2:**
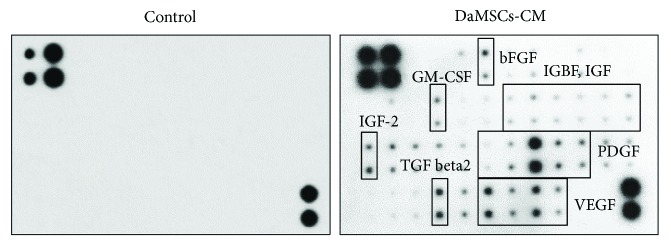
Growth factor secretion profiles of DaMSC-conditioned media (DaMSC-CM). A human growth factor antibody array was used to detect paracrine factors in DaMSC-CM. DMEM was used as a negative control.

Recently, active biomolecules secreted from stem cells have been considered to have benefits in tissue regeneration because secreted molecules such as cytokines, growth factors, and extracellular matrix (ECM) molecules can contribute to the host tissue's microenvironment. Thus, we tested the paracrine effects of stem cells using a growth factor chip array. After treating media with DaMSCs, 26 secreting growth factors were detected in the conditioned media (Figure 2 and Supplementary Table S2). Normal media was used for a negative control for the chip array. Among growth factors, basic fibroblast growth factor (bFGF), insulin-like growth factor binding protein (IGFBP), insulin-like growth factors (IGFs), granulocyte macrophage colony-stimulating factor (GM-CSF), platelet-derived growth factors (PDGFs), vascular endothelial growth factors (VEGFs), and transforming growth factor-*β*2 (TGF-*β*2) were discovered. In particular, PDGF, VEGF, and TGF-*β*2 were significantly released from DaMSCs.

### 3.3. Effects of DaMSC-CM on PDC Proliferation

**Figure 3:**
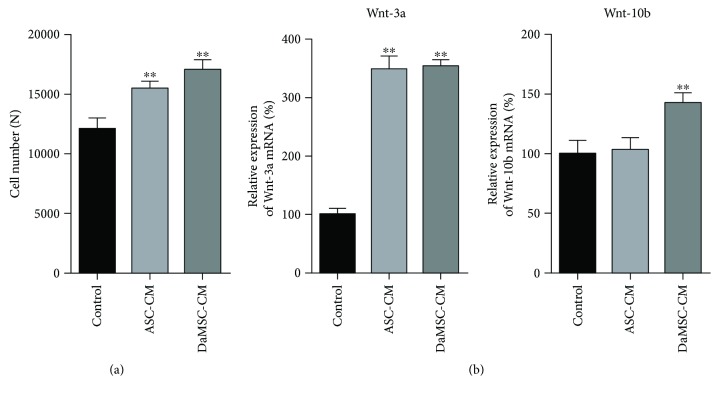
Effects of hair cell development on treatment with DaMSC-CM. (a) Proliferation of dermal papilla cells (DPCs) treated with control, adipose tissue-derived stem cell conditioned media (ASC-CM), or DaMSC-CM for 48 h. (b) Activation of Wnt signaling in DPCs treated with control, ASC-CM, and DaMSC-CM. qRT-PCR analysis exhibits relative expression of Wnt-3a mRNA (b, left) and Wnt-10b mRNA (b, right), normalized to expression of GAPDH mRNA. Data represent the mean ± s.d. (*n* = 3). ^∗∗^ *P* < 0.001 by one-way analysis of variance with Tukey's multiple comparison tests, as compared with the control.

Mesenchyme-derived dermal papilla cells (DPCs), located at the bottom of the hair follicle, play important roles in regulating hair follicle generation [25, 26]. Hair follicle development is deeply associated with signaling between epithelial keratinocytes and DPCs [27]. In particular, Wnt/*β*-catenin signaling is required for the hair-inducing activity of dermal papillae, which is crucial for the proliferation of DPCs [28]. Thus, we first tested whether DaMSC-CM can induce DPC proliferation (Figure 3(a)). DaMSC-CM was used to treat DPCs for 48 h and then cell proliferation was evaluated using a CCK-8 assay. We also used adipose tissue-derived stem cell-conditioned media (ASC-CM) for comparison. Interestingly, DaMSC-CM promoted significantly more cell growth than ASC-CM. Additionally, DaMSC-CM showed almost 1.4-fold increase in cell number compared to normal media. Next, Wnt signaling seems to play important roles in the mechanisms of tissue repair and regeneration in both fetal and adult wounds [29, 30]. The Wnt pathway also regulates cell proliferation in wound healing. In particular, skin wounds express various Wnt proteins with transcripts of Wnt-1, -3a, -4, -5a, and -10b [31]. In addition, hair follicle regeneration and MSC activation can be induced via activation of Wnt/*β*-catenin signaling pathway regarding to Wnt-3a and Wnt-10b [32]. Thus, Wnt proteins such as Wnt-3, Wnt-10a, and Wnt-10b play a key role in hair follicle initiation, morphogenesis, and development [33]. To study the effect of Wnt signaling on hair growth, expression of Wnt-3a and Wnt-10b was analyzed using quantitative RT-PCR (Figures 3(b) and 3(c)).

Treatment with DaMSC-CM led to a 3.2-fold increase in Wnt-3a mRNA and 1.5-fold increase in Wnt-10b mRNA compared to normal media. Therefore, DaMSC-CM promotes activation of Wnt signaling pathways for hair regeneration.

### 3.4. Effects of DaMSC-CM on Skin Regeneration

**Figure 4:**
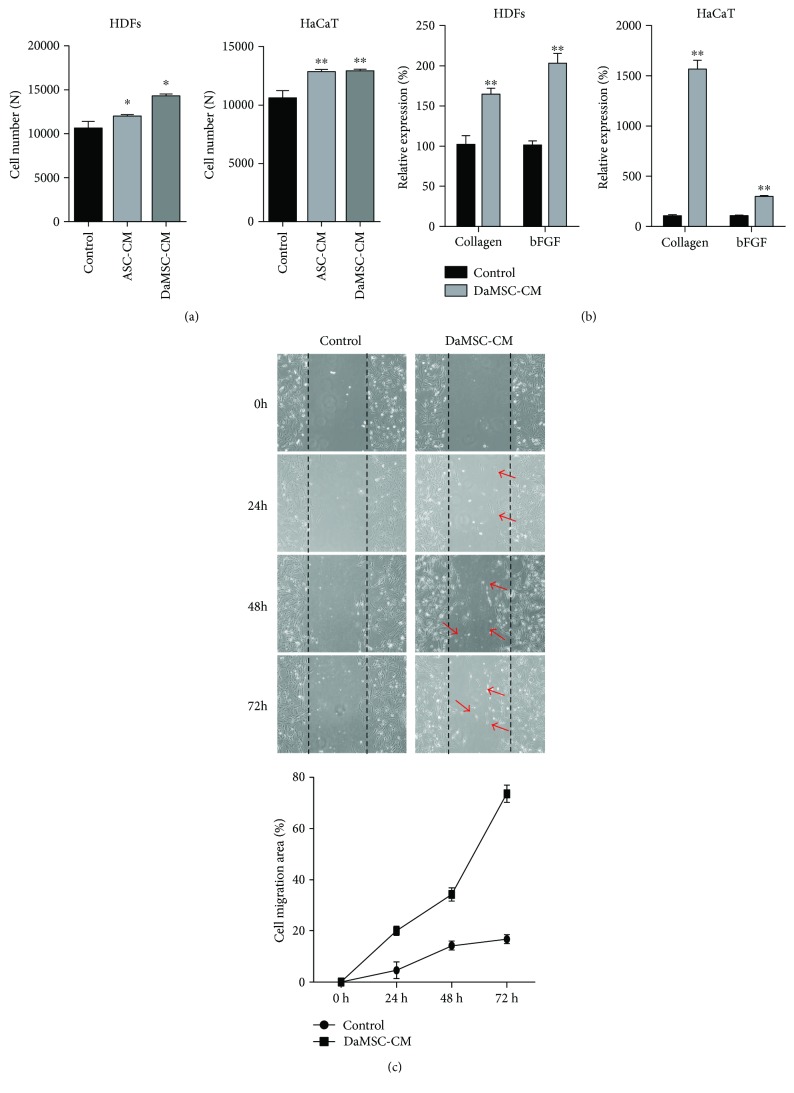
Effects of skin regeneration on treatment with DaMSC-CM. (a) Proliferation of human dermal fibroblasts (HDFs) and keratinocytes (HaCaTs) under treatment of three different types of media for 48 h. (b) qRT-PCR analysis showing relative expression of collagen type Ι mRNA (b, left) and basic fibroblast growth factor (bFGF) mRNA (b, right), normalized to expression of GAPDH mRNA. (c) *In vitro* wound healing assay exhibiting HDF migration upon treatment with DaMSC-CM in a time-dependent manner. Red arrows indicate cell migration. Black lines represent wound spaces created with plastic micropipette tips. Data represent the mean ± s.d. (*n* = 3). ^∗^ *P* < 0.01, ^∗∗^ *P* < 0.001 by one-way analysis of variance with Tukey's multiple comparison tests, as compared with the control.

Several studies have revealed that stem cells closely contribute to wound repair after damage, resulting in regeneration of damaged skin [34, 35]. Thus, we tested the effects of DaMSC-CM on wound healing. First, we investigated the proliferation of skin-related cells including keratinocytes (HaCaTs) and human dermal fibroblasts (HDFs) upon treatment with DaMSCs (Figure 4(a)). Compared to normal media, DaMSC-CM enhanced proliferation of both types of skin cells. In addition, we investigated the expression of skin regeneration-related genes after treatment with DaMSC-CM (Figure 4(b)). Remarkably, DaMSC-CM treatment led to high levels of collagen type 1 and bFGF mRNA compared with normal media. Furthermore, to test the effects of DaMSC-CM on cell migration, a time-dependent *in vitro* wound healing assay was carried out (Figure 4(c)). Similar to our findings of cell proliferation, we observed enhanced migration of HDFs after artificial scratches of a confluent cell monolayer when treated with DaMSC-CM, especially at 72 h posttreatment, when the migration rate of cells treated with DaMSC-CM was approximately 75%. Thus, DaMSC-CM also has regenerative effects on skin wounds.

### 3.5. Isolation of Secretory Vesicles from DaMSC-CM as Mediator of Paracrine Actions

**Figure 5:**
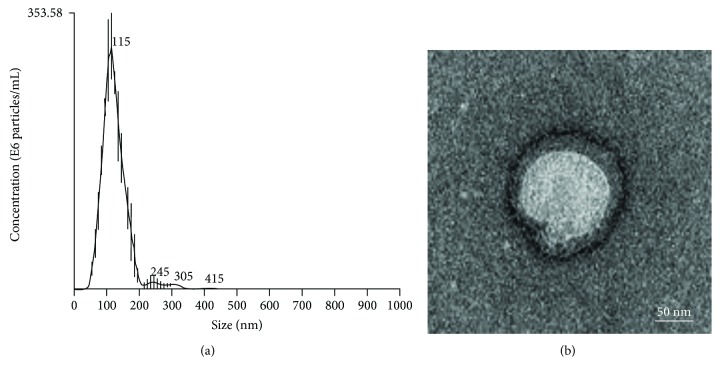
Characterization of extracellular vesicles (EVs) derived from DaMSCs. (a) Nanoparticle tracking analysis (NTA) showed a size distribution of EVs with an average of 119.9 nm. (b) Transmission electron microscopic (TEM) images revealed the morphology of the EVs with a membrane bilayer. Scale bar indicates 100 nm. Particle number: 2.29 × 10^15^ particles/mL.

**Figure 6:**
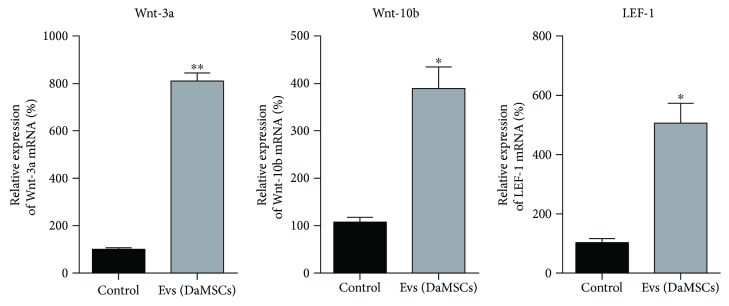
Activation of skin regeneration-related genes upon treatment with EVs. qRT-PCR analysis was conducted after treatment of DPCs with EVs alone. DMEM treatment was used as a negative control. mRNA expression of Wnt-3a, Wnt-10b, and lymphoid enhancer-binding factor-1 (LEF-1) was normalized to the expression of GAPDH mRNA. Data represent the mean ± s.d. (*n* = 3). ^∗^ *P* < 0.01, ^∗∗^ *P* < 0.001 by one-way analysis of variance with Tukey's multiple comparison tests, as compared with the control.

Recent studies reported that EVs from stem cells may contribute to paracrine signaling [19, 36, 37]; therefore, we tried to purify secretory vesicles from DaMSC-CM (Figure 5). Surprisingly, many vesicles were detected when isolated from DaMSC-CM using an ultracentrifugation approach. NTA analysis obtained EVs with a size distribution of average 119.9 nm (Figure 5(a)), and TEM images exhibited nano-sized vesicles with lipid bilayers (Figure 5(b)). Next, to investigate the potential function of EVs isolated from DaMSC-CM on tissue regeneration, gene expression related to Wnt signaling pathways and hair follicle formation were evaluated after treatment of DaMSC-CM with EVs (Figure 6). Interestingly, EVs alone highly increased mRNA levels of Wnt-3a and Wnt-10b ~8-fold and ~4-fold, respectively. In contrast, our previous data showed that DaMSC-CM induced only 3.2-fold and 1.5-fold increases in the mRNA levels of Wnt-3a and Wnt-10b (Figure 3(b)). We assumed that EVs isolated from DaMSCs are highly enriched with growth factors and other factors. In addition, the level of LEF1 mRNA, which is a major endpoint mediator of the Wnt signaling pathway [38], was significantly increased under the treatment with EVs 5.6-fold (Figure 6). Thus, DaMSCs secrete extracellular vesicles that can act as mediators of paracrine actions.

## 4. Discussion

Stem cells have great potential to differentiate and regenerate into all types of cells and are thus unique cells in the body. Stem cell-based therapies have been conducted in auto- and allogenic transplantation for reconstruction of tissues. Many molecular components contribute to regenerative processes and have influences on engraftment [39, 40]. Stem cells with the ability for homing and self-renewal play crucial roles in mechanisms of tissue regeneration, but paracrine actions are also essential for restoration of tissues. In addition to their multipotent differentiation potential, stem cells can release cytokines, chemokines, and growth factors after injury. These paracrine factors can influence adjacent cells and their microenvironments. Interestingly, paracrine mediators seem to be expressed in response to injury. It had previously been reported that intravenous and intracoronary injection of MSCs-CM significantly restored ventricular performance in an injury model [41]. Notably, nanovesicles in CM were discovered with a range of 100–220 nm in diameter, suggesting that paracrine signaling may be affected by EVs acting as mediators between cells. Several approaches, including proteomics, chip arrays, microarrays, and other high-throughput analyses, have been performed to identify the content of vesicles [17]. Recently, many studies have discovered the function of MSC-derived exosomes on therapeutic effects [42]. Secretary vesicles including exosomes and microvesicles are involved in direct or indirect cell-to-cell communication, thereby altering tissue environment and metabolism. These vesicles carry diverse cytokines and growth factors, lipids, small RNAs, and so on, which can influence tissue responses to injury, disease, and infection. Surprisingly, MSCs and MSC-derived exosomes exhibit similar therapeutic effects in sparing tissue or promoting regeneration from injury sites [43, 44]. Thus, MSC-derived exosomes have great merit in cell-free therapeutics.

In our study, we also revealed that DaMSCs released high levels of soluble factors that may induce activation of canonical Wnt/*β*-catenin signaling. The EVs that we found in this study may also influence tissue regeneration as paracrine mediators. Accordingly, it has been proposed that Wnts are secreted on exosomes and contribute to tissue injury repair [45, 46]. Thus, our investigation of DaMSC-derived EVs acting in tissue regeneration gives rise to a new aspect of regenerative medicine in which EVs may be a unique material for rapid and complete tissue regeneration.

## 5. Conclusions

In this study, we demonstrated that DaMSC-CM can significantly promote tissue regeneration such as hair follicle formation and skin wound healing, which are both associated with Wnt signaling pathways. DaMSCs can release bioactive molecules including PDGF, VEGF, and TGF-*β*2 as paracrine factors for cell-to-cell communication. Furthermore, we identified nano-sized vesicles derived from DaMSC-CM and investigated their ability to promote hair follicle formation via Wnt signaling. These results suggested that secretory vesicles act as paracrine mediators for regulation of cellular responses. Thus, our findings indicate great potential for application in regenerative medicine.

## Supplementary Materials

Supplementary Materials Table S1: a list of primers for qRT-PCR analysis. The relative expression of each mRNA was analyzed using qRT-PCR and normalized by the expression of GAPDH mRNA. Table S2: a list of secreted growth factors. The paracrine effects of stem cells using a growth factor chip array were tested.
