# Supplementary material for: A Novel Secretory Vesicle from Deer Antlerogenic Mesenchymal Stem Cell-Conditioned Media (DaMSC-CM) Promotes Tissue Regeneration
Source: Stem Cells Int. 2018 Mar 25;2018:3891404. doi: 10.1155/2018/3891404 (PMC5889873; doi:10.1155/2018/3891404)
Supplement: Supplementary Materials — Table S1: a list of primers for qRT-PCR analysis. The relative expression of each mRNA was analyzed using qRT-PCR and normalized by the expression of GAPDH mRNA. Table S2: a list of secreted growth factors. The paracrine effects of stem cells using a growth factor chip array were tested. [file 3891404.f1.pdf]

## Supporting information

Table S.1. A list of primers

| Primer | | oligo sequence |
|---|---|---|
| Wnt10b | Forward | 5'-TGGAAGAATGCGGCTCTGA-3' |
| | Reverse | 5'-CTCTCCAAAGTCCATGTCATGG-3' |
| Wnt3a | Forward | 5'-TGTTGGGCCACAGTATTCCT-3' |
| | Reverse | 5'-ATGAGCGTGTCACTGCAAAG-3' |
| GAPDH | Forward | 5'-CCTGACCTGCCGTCTAGAAA-3' |
| | Reverse | 5'-TGACAAAGTGGTCGTTGAGG-3' |
| COL1 | Forward | 5'-GGAGGAGAGTCAGGA-3' |
| | Reverse | 5'-GCAACACAGTTACAC-3' |
| bFGF | Forward | 5'-AGGAGTGTGTGCTAACCGTT-3' |
| | Reverse | 5'-CAGTTCGTTTCAGTGCCACA-3' |

Table S.2. A list of secreting growth factors

| | | | | | | | | | | | |
|---|---|---|---|---|---|---|---|---|---|---|---|
| POS | POS | NEG | NEG | AR | bFGF | Beta-NGF | EGF | EGFR | FGF-4 | FGF-6 | FGF-7 (KGF) |
| GCSF | GDNF | GM-CSF | HB-EGF | HGF | IGFBP-1 | IGFBP-2 | IGFBP-3 | IGFBP-4 | IGFBP-6 | IGF-1 | IGF-1 R |
| IGF-2 | M-CSF | M-CSF R | NT-3 | NT-4 | PDGF R alpha | PDGF R beta | PDGF-AA | PDGF-AB | PDGF-BB | PLGF | SCF |
| SCF R (CD117) | TGF alpha | TGF beta 1 | TGF beta 2 | TGF beta3 | VEGF-A | VEGFR2 | VEGFR3 | VEGF-D | BLANK | BLANK | POS |
